# A rare case of bevacizumab-related osteonecrosis of the jaw associated with dental implants

**DOI:** 10.1186/s40729-019-0188-0

**Published:** 2019-10-01

**Authors:** Gustavo Maluf, Rogério Jardim Caldas, Eduardo Rodrigues Fregnani, Paulo Sérgio da Silva Santos

**Affiliations:** 1 Private Clinic, Brasília, DF Brazil; 2 Brasília, Brasil; 3 Department of Stomatology, Bauru Dental School, USP–University of São Paulo, Bauru, SP Brazil; 4 Department of Oral Medicine, Hospital Sírio-Libanês, São Paulo, SP Brazil; 5 Department of Stomatology, Bauru Dental School, USP–University of São Paulo, Bauru, SP Brazil

**Keywords:** Osteonecrosis, Jaw, Bevacizumab, Angiogenesis inhibitors

## Abstract

**Background:**

Medication-related osteonecrosis of the jaw (MRONJ) is characterized by the development of bone necrosis in the jaws of patients receiving antiresorptive and/or antiangiogenic medications. No scientific reports have been published yet on bevacizumab-related osteonecrosis of the jaw (BeRONJ) when associated with dental implant placement and adjuvant ozone therapy.

**Case presentation:**

A 54-year-old female patient with a history of metastatic breast cancer and bevacizumab use presented with a dental infection. Dental extraction followed immediately by dental implant placement was planned after suspension of the bevacizumab treatment. The patient presented with pain, drainage of purulent secretion, and bone exposure 5 weeks post-surgery. Complete healing was achieved at postoperative 7 months.

**Conclusions:**

The combination of adjuvant ozone therapy and surgical debridement was effective for the treatment of MRONJ; however, the risk of MRONJ may persist after the suspension of bevacizumab for 28 days.

## Background

The treatment of malignancies often involves the use of targeted therapies to control the growth and survival of malignant cells by interfering with specific molecular agents involved in carcinogenesis [1]. Bevacizumab is a recombinant humanized monoclonal antibody designed to selectively bind and inhibit the biological activity of all human vascular endothelial growth factor (VEGF-A) isoforms. It is mainly used for the treatment of advanced cancers, such as metastatic colon, kidney, brain, and lung cancer [2].

Antiangiogenic agents may increase the risk of medication-related osteonecrosis of the jaw (MRONJ), either as monotherapy or in combination with bisphosphonates [3]. These drugs compromise microvascular integrity and may lead to subclinical jawbone involvement. Moreover, the ability of these drugs to impair angiogenesis explains how bevacizumab can lead to the collapse of the oral mucosa and subsequent bone exposure [4].

Dental implant placement may precipitate MRONJ in patients exposed to bisphosphonates [5–7]. Although Greuter et al. in 2008 [8] have reported a case of osteonecrosis of the jaw in a patient receiving bevacizumab therapy after dental extraction, to our knowledge, no similar cases involving dental implants have been previously described. According to the American Association of Oral and Maxillofacial Surgery, dental implant placement should be avoided in oncologic patients receiving antiresorptive therapy or antiangiogenic medications, although antiresorptive therapy for osteoporosis is not an absolute contraindication for this surgical procedure [9]. In another study, dental implants were not recognized as a risk factor for MRONJ in patients receiving denosumab, despite the fact that dental implant placement is inadvisable for cancer patients [10].

Many clinicians are aware of the associated risk of MRONJ around dental implants in patients exposed to bisphosphonates; however, a consensus regarding the use of bevacizumab is still lacking in the literature. Thus, the purpose of this manuscript was to report a rare case of osteonecrosis of the jaw related to the use of bevacizumab in a patient who received dental implants.

## Case presentation

**Fig. 1:**
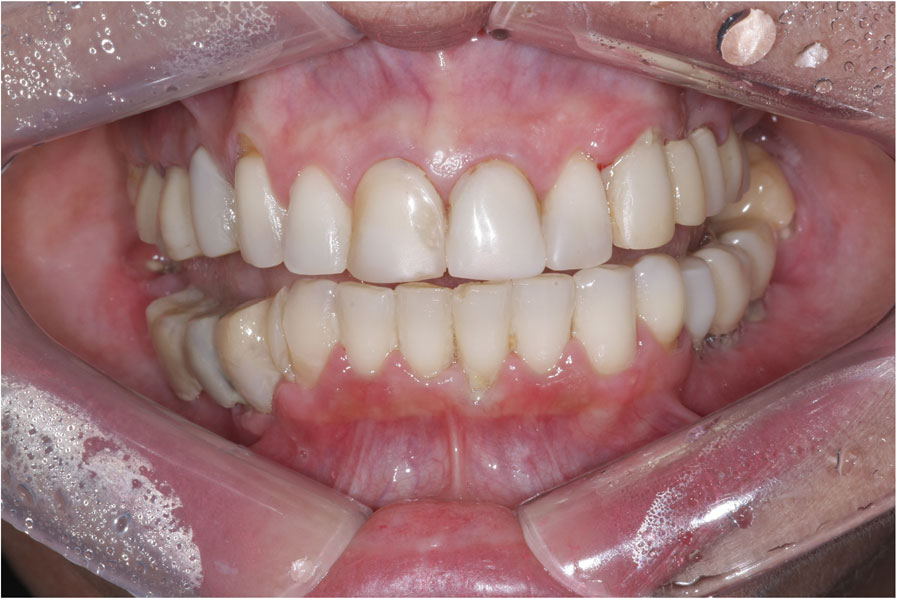
Initial clinical image showing oral infection foci

**Fig. 2:**
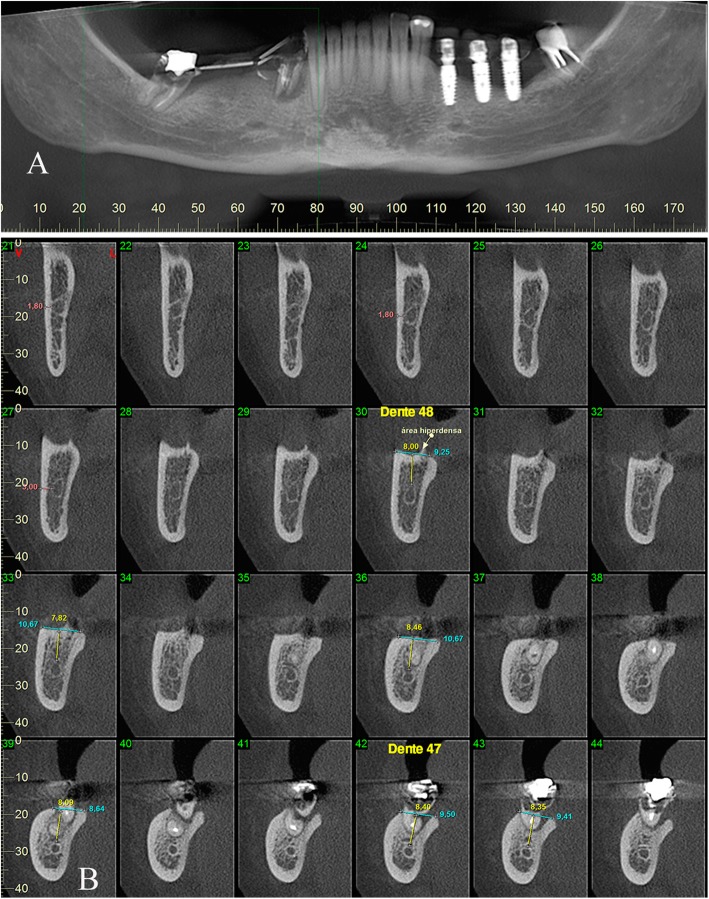
**a**, **b** Tomographic findings: lesions, measurements, and bone quality

**Fig. 3:**
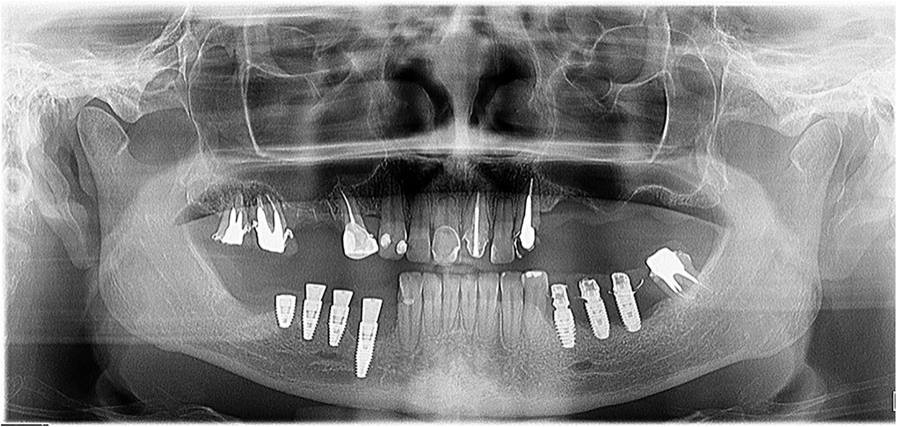
Panoramic radiograph after dental extractions with subsequent dental implants

A 54-year-old Caucasian woman complained of an unpleasant taste and pain in the mouth. Her medical history included breast cancer with metastasis, which was diagnosed in 2007. The patient had no comorbidities and no history of smoking. A radical mastectomy with axillary dissection was performed. The patient had no previous history of radiotherapy of the head and neck or use of bisphosphonates. The patient received bevacizumab (400 mg/16 mL every 2 weeks; 32 infusions in total) from 11 April 2014 to 26 October 2016. Docetaxel (30 mg/m^2^ on D1 and D15 of the cycle) and carboplatin (386 mg on D1 and D15 of the cycle) infusions were started in April 2014 and suspended in September 2016. The patient’s leukocyte count was 4310 cells/mm^3^ (segmented neutrophils, 1896/mm^3^; band neutrophils, 0/mm^3^). At 28 days following suspension of the cancer treatment, intraoral clinical examination revealed drainage of purulent secretion involving teeth 16, 25, 27, 44, and 47 (Fig. 1). Cone-beam computed tomography (CT) showed the association of hypodense areas with the remaining roots of teeth 16, 25, and 27, and disruption of the lower cortical regions of the maxillary sinus. Hypodense areas could also be seen associated with the roots of teeth 44 and 47 (Fig. 2). The patient did not present with clinical characteristics or radiographic findings to suggest MRONJ, and dental implant was placed on 19 December 2016 (3 months after suspension of her medication). At 54 days after the last dose of bevacizumab, and on completion of 3 months of docetaxel and carboplatin infusions, debridement and dental extractions of teeth 16, 25, 27, 44, and 47 were performed in combination with immediate insertion of Straumann® Bone Level Tapered-BLT® implants (SLActive) in regions of teeth 44, 45, 46, and 47 (Fig. 3). Chlorhexidine 0.12% mouth wash and levofloxacin (Levoxin®) were prescribed 5 days before and after the oral implantation surgery and continued 5 days after the implantation surgery.

**Fig. 4:**
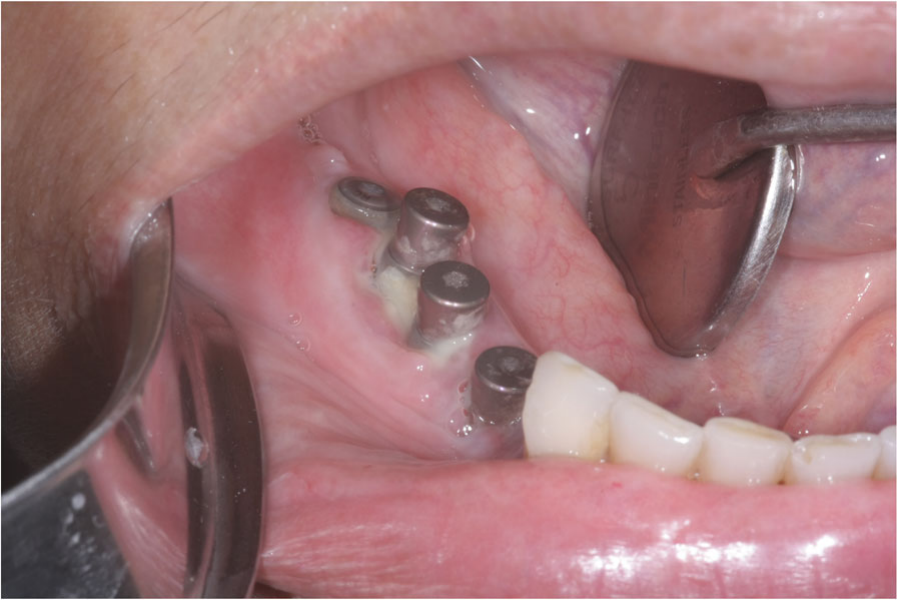
Bone exposure surrounding the implants with drainage of purulent secretion

Five weeks postoperatively, pain, drainage of purulent secretion, and bone exposure around the implants were observed (Fig. 4), although none of the implants showed mobility. Ten ozone therapy sessions associated with levofloxacin were performed. After 4 weeks of therapy with ozone oil (Philozon®, Balneário Camboriú, SC, Brazil), no pain or drainage of purulent secretion were present. Bone sequestration accompanied by implant mobility could, however, be seen. Local debridement and implant removal were performed.

**Fig. 5:**
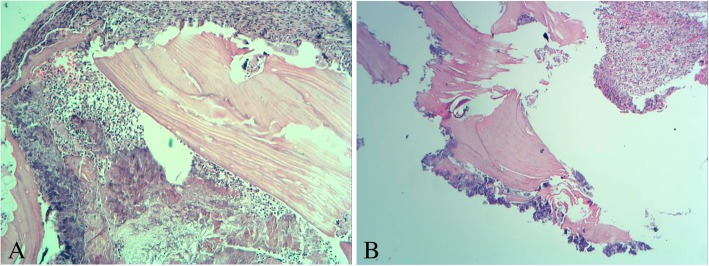
**a**, **b** Histological image of the lesion

The material removed consisted of 3 irregular fragments of hard, brown bone tissue, with the largest measuring 1.0 × 1.0 × 0.3 cm and the smallest measuring 0.4 × 0.4 × 0.3 cm. The material was stained with hematoxylin and eosin, and histological sections revealed irregular fragments consisting of devitalized bone and the presence of osteoclasts. Adjacent to the necrotic trabeculae, fibrous connective tissue exhibiting intense mixed inflammatory infiltrates (neutrophils, lymphocytes, plasma cells, and some macrophages) was found. Bacterial colonies and hemorrhagic foci were also noted (Fig. 5). Based on these results, the histological examination indicated osteonecrosis, categorized as stage 2 MRONJ [9].

**Fig. 6:**
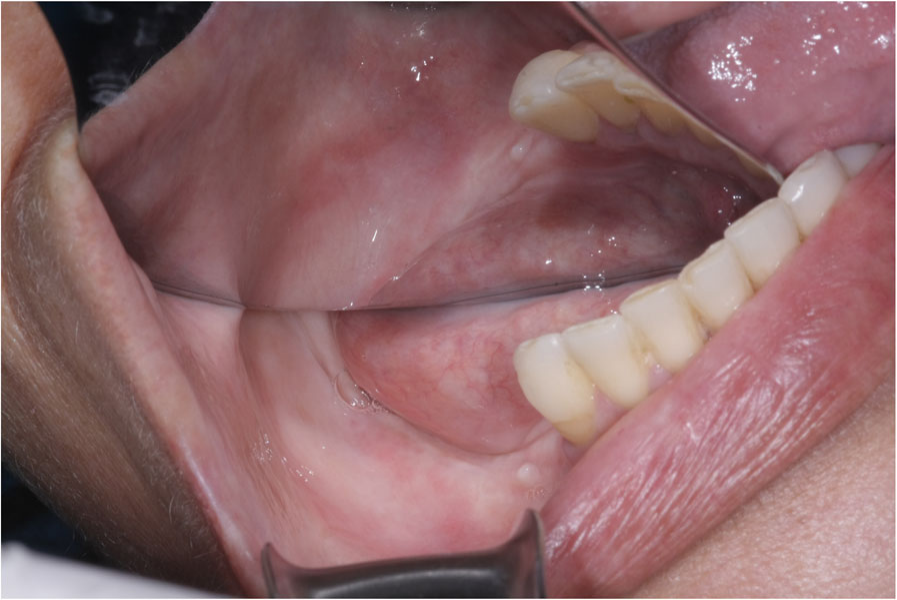
Image obtained 7 months postoperatively showing no lesions

**Fig. 7:**
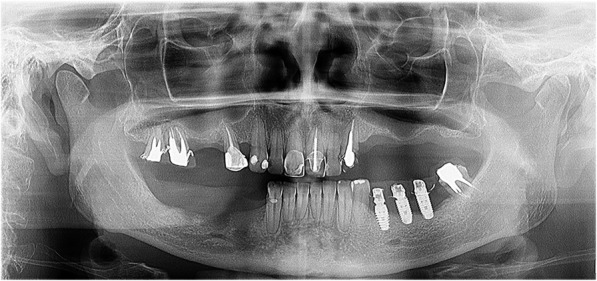
Panoramic radiograph at 7-month follow-up

At 14 days after debridement, the alveolar ridge was completely covered by soft tissue without bone exposure, and the patient was no longer experiencing pain. In March 2017, after the resolution of the osteonecrosis, the patient underwent the same chemotherapy regimen (bevacizumab, carboplatin, and docetaxel) as administered earlier. In July 2017, the patient was still undergoing chemotherapy. At the 7-month postoperative follow-up, the debrided area presented a healthy mucosal covering without lesions (Fig. 6). No signs of bone lysis or sequestration were seen on the panoramic radiograph (Fig. 7).

## Discussion

A meta-analysis showed that the placement of dental implants in patients exposed to bisphosphonates did not reduce the success rate of the dental implant [11], although these patients may experience complications. Based on this analysis, a risk assessment should be performed on a case-by-case basis, since MRONJ is rare but one among serious complication of bisphosphonate therapy [11]. However, a case-control study showed a significant negative influence of bisphosphonates on the success rate of dental implants [12]. In fact, the placement of dental implants is often considered a risk factor for the development of MRONJ [7, 10]. The cases of bevacizumab-related osteonecrosis of the jaw (BeRONJ) reported in the literature presented different triggering factors, such as dental extraction or denture trauma [4, 8, 13]. To the best of our knowledge, there is no data on osteonecrosis involving implant placement in patients who have received antiangiogenic drugs. Therefore, this report presents a rare case of BeRONJ related to the placement of dental implants.

The success rates of dental implants immediately inserted into the sockets of teeth with periodontal and periapical lesions are similar to those in cases without infection, provided that local debridement, meticulous cleaning of the tooth socket, mouth-washing with chlorhexidine, and pre- and postoperative systemic antibiotic use are performed [14, 15]. In our case report, the patient presented with infection involving both the superior and inferior teeth. Nonetheless, the insertion of dental implants was only planned for the mandible, as bone graft use was indicated before the placement of the superior dental implants. Considering that the patient would go back to receiving chemotherapy and the associated improvement in patient’s quality of life, a single-stage surgical procedure (dental extraction immediately followed by implant insertion) was considered a feasible approach for the mandible. In order to control local infection and decrease the risk of complications, an oral antibiotic was prescribed 5 days before the insertion of the dental implants, and the aforementioned measures were implemented.

Long-term administration of antiangiogenic agents does not directly indicate a high risk of MRONJ [16], though some authors have established 7-, 14-, and 28-day intervals of bevacizumab suspension for oral surgery [17–19]. In this case, the antiangiogenic medication was suspended for 28 days before implant placement; however, this was not enough to prevent MRONJ. The surfaces of Straumann® SLactive implants have been shown to present rapid osseointegration occurring between 21 and 28 days before implantation [20]. In order to avoid interference with the bevacizumab regimen and to accelerate osseointegration, Straumann® Bone Level Tapered-BLT® implants (SLactive) were used in this case. Of the four inserted implants, three were osseointegrated by the time that osteonecrosis manifested. Kwon et al., in 2014 [7], also reported bone sequestration involving histologically osseointegrated implants. On the other hand, the formation of sequestra may still continue in the deep regions of cancellous bone.

August et al. [21] reported in 2002 that the survival rate of dental implants is not significantly impacted by chemotherapy, regardless of whether administered before or after implant insertion. In contrast, Dantas et al. [22] reported that a 30-day period of chemotherapy suspension for implant placement would negatively affect osseointegration. Therefore, a consensus could not be established regarding the exact safe period for dental implant placement in patients undergoing chemotherapy. In this case, docetaxel and carboplatin infusions had been already suspended for 3 months preoperatively and resumed 3 months postoperatively. Additionally, these infusions do not represent a risk factor for MRONJ, according to Ruggiero et al. [9].

In addition to the risk of MRONJ for oral surgery, oral function and quality of life also play important roles in deciding the indications for a dental surgical procedure on a patient receiving antiresorptive agents [23]. To the best of our knowledge, no contraindication for dental implant installation in patients exposed to bevacizumab has been reported in scientific literature, when a period of 28 days without medication is observed pre-and post-procedure [17–19]. Nonetheless, specialists should be aware of the complexity of managing such patients.

MRONJ is a side effect of drugs with a predilection for the mandible [24], which can be attributed to the relatively low vascularization of the mandible as compared to the maxilla [13]. Dental infections, surgical interventions, corticosteroid treatment, and chemotherapy have been described as risk factors for osteonecrosis [25]. In this case, the patient presented with stage 2 osteonecrosis with dental infections in an area of the mandible, even after receiving antibiotic therapy, prior to implant placement.

The treatment of MRONJ with ozone therapy in conjunction with debridement and antibiotics presented a success rate of 90% in 131 patients with a history of bisphosphonate use [26]. Ozone therapy is occasionally beneficial, including antimicrobial, fungicidal, and virucidal activities [27, 28]; stimulation of the vascular system which increases tissue oxygenation; modulation of the immune response; stimulation of angiogenesis; and replication of fibroblasts [29]. Ozone induces bone sequestration, which is a favorable prognostic sign; because the necrotic bone tissue is removed during debridement, a bleeding bone with well-vascularized mucosa can be observed underneath [23]. Similar findings were noted in our case. After 10 sessions of ozone therapy, bone sequestration and loss of dental implants occurred, revealing the remaining vascularized alveolar bone.

## Conclusions

The findings of the present case indicate that insertion of dental implants is a risk factor for osteonecrosis of the jaw in patients exposed to bevacizumab. Therefore, prospective randomized studies should be encouraged to determine a safer bevacizumab regimen that considers both oral surgeries and the risk of dental implants for the management of osteonecrosis in clinical settings.

## Data Availability

Data sharing is not applicable to this article as no datasets were generated or analyzed during the current study.

## Abbreviations

BeRONJ: Bevacizumab-related osteonecrosis of the jaw
BLT: Bone level tapered
CT: Computed tomography
MRONJ: Medication-related osteonecrosis of the jaw
VEGF-A: Vascular endothelial growth factor A
